# Supplementary material for: Dynamic microvilli sculpt bristles at nanometric scale
Source: Nat Commun. 2024 May 13;15:3733. doi: 10.1038/s41467-024-48044-3 (PMC11091046; doi:10.1038/s41467-024-48044-3)
Supplement: Supplementary file 1 — Supplementary Information [file 41467_2024_48044_MOESM1_ESM.pdf]

Supplementary information

Dynamic microvilli sculpt bristles at nanometric scale

Kyojiro N Ikeda, Ilya Belevich, Luis Zelaya-Lainez, Lukas Orel, Josef Füssl, Jaromír Gumulec, Christian Hellmich, Eija Jokitalo, Florian Raible

Supplementary information includes:

Supplementary Figures 1 to 5

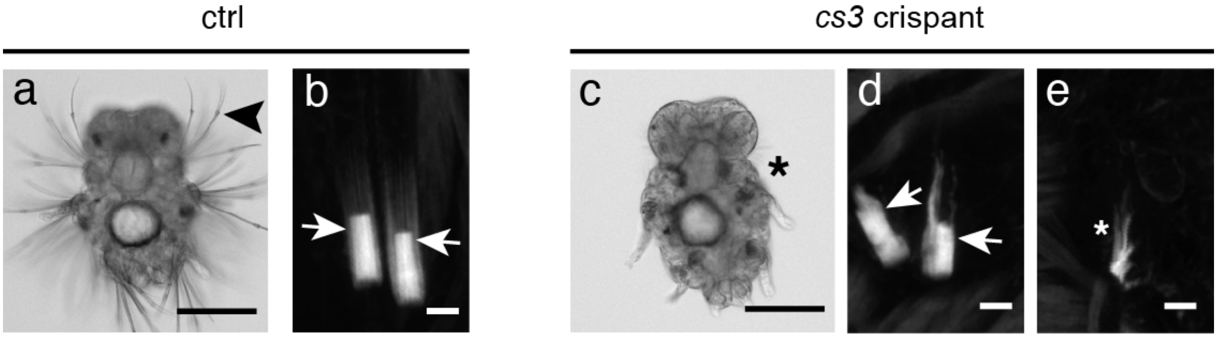

Supplementary Fig. 1 | Formation of microvilli in *cs3* crispants. (a, b) Overall morphology (a) and corresponding stage III microvillar pattern (b) of a control-injected individual (72 hpf); arrowhead in (a) points at a regular bristle; arrows in b demarcate the diagnostic microvillar assembly of the stage III chaetoblast **(c, d, e)** A *cs3* crispant (c) lacking bristles (asterisk), and corresponding microvillar patterns (d, e) exhibiting irregularities; patterns in (d) reveal a broader structure reminiscent of the diagnostic axial microvillus (arrows); patterns in (e) (marked by asterisk) are different from the canonical patterns in Fig. 2 b-g. Scale bars: 100 µm (a, c), 2µm (b, d, e).

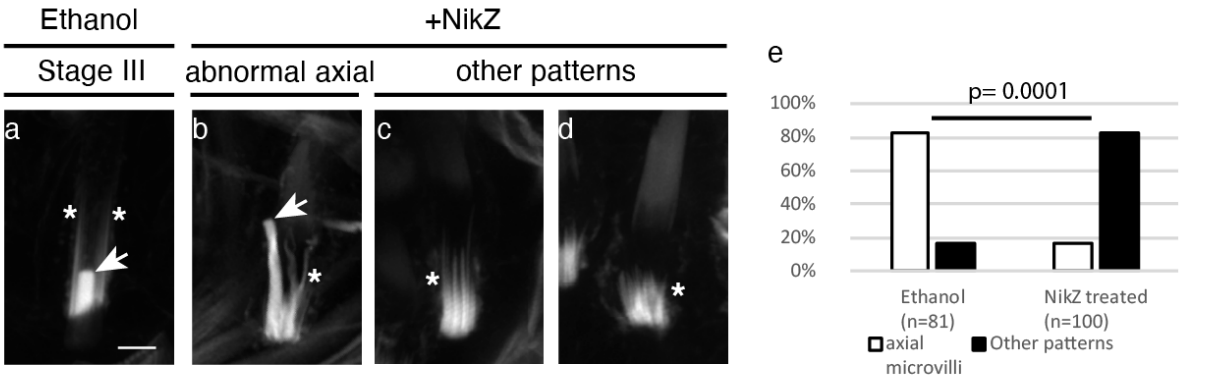

Supplementary Fig. 2 | Formation of microvilli upon inhibition of chitin synthesis with NikZ. (a) Regular stage III pattern of microvilli observed after treating animals from 24hpf to 64hpf with the vehicle (0.1% ethanol); arrow: axial microvillus; asterisks: ancillary microvilli. **(b-d)** Microvillar patterns observed after treatment with NikZ: (b) pattern including a wider microvillus resembling an axial microvillus (arrow), but slimmer markedly longer, (c) ordered pattern with parallel microvilli, (d) disordered microvilli. **(e)** Fractions of patterns containing at least one microvillus with an apical tip broader than 300nm; comparison between ethanol-treated larvae (left) and NikZ-treated larvae (right), revealing a significant difference between the conditions (p value: Chi-square test). Scale bar: 2 µm.

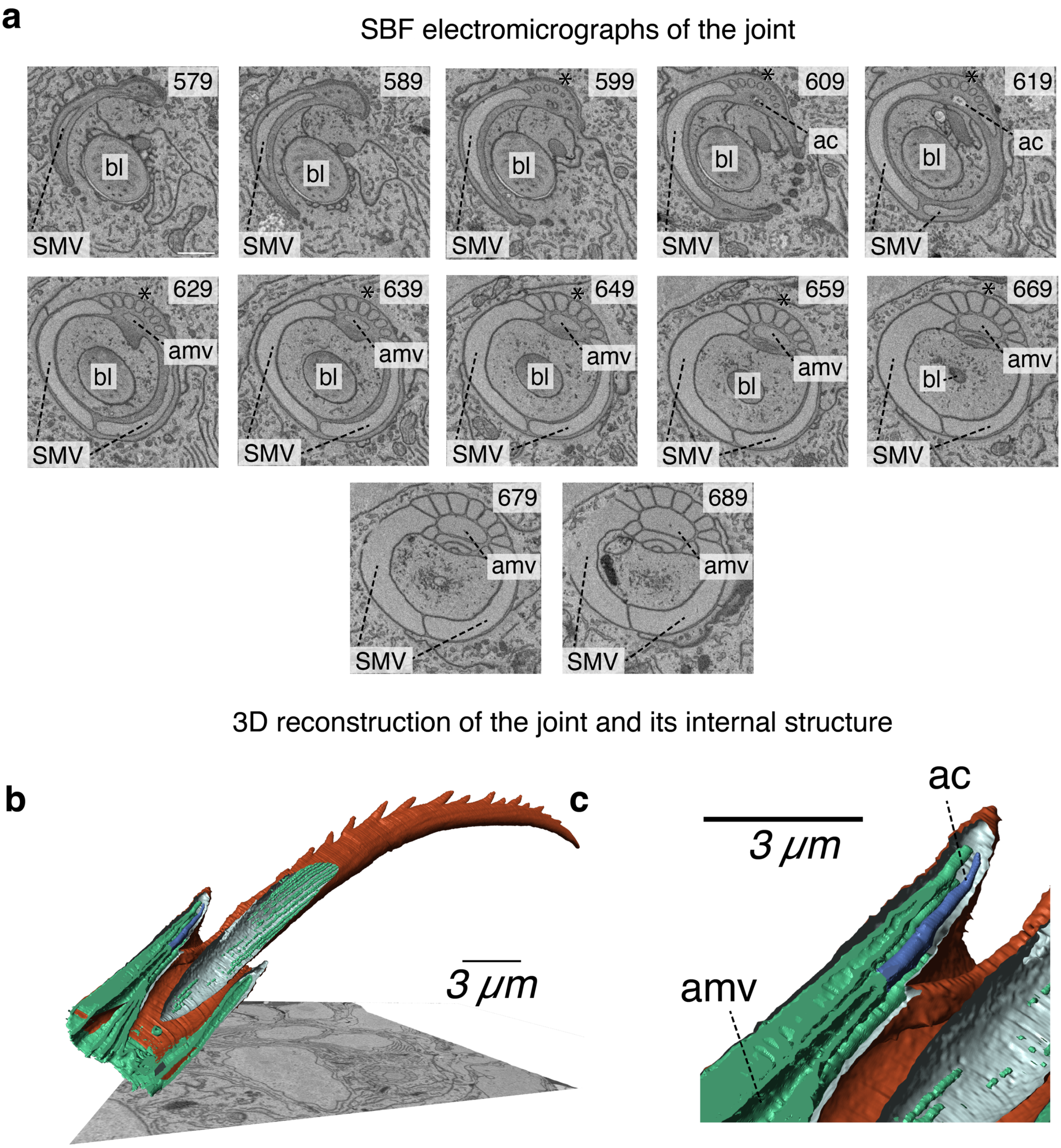

Supplementary Fig. 3 | Joint geometry reveals the origin of the axial microvillus in the boss of the joint. (a) Selected SBF electronmicrographs of the joint. Panels correspond to the indicated ~40nm cross-sections / frames of the shaft in Supplementary Video 4 and are sorted in the order of their occurrence from the distal (579) to proximal (689) direction. Continuity of the axial channel (ac) / axial microvillus (amv) across sections demonstrates the origin of relevant shaft geometry in the boss of the joint. Size bar is 1 μm. **(b, c)** Overview and details of the resulting 3D reconstruction, bl: blade; ac: axial channel; amv: axial microvillus; smv: semicircular microvillus. Asterisks demarcate the boss of the joint.

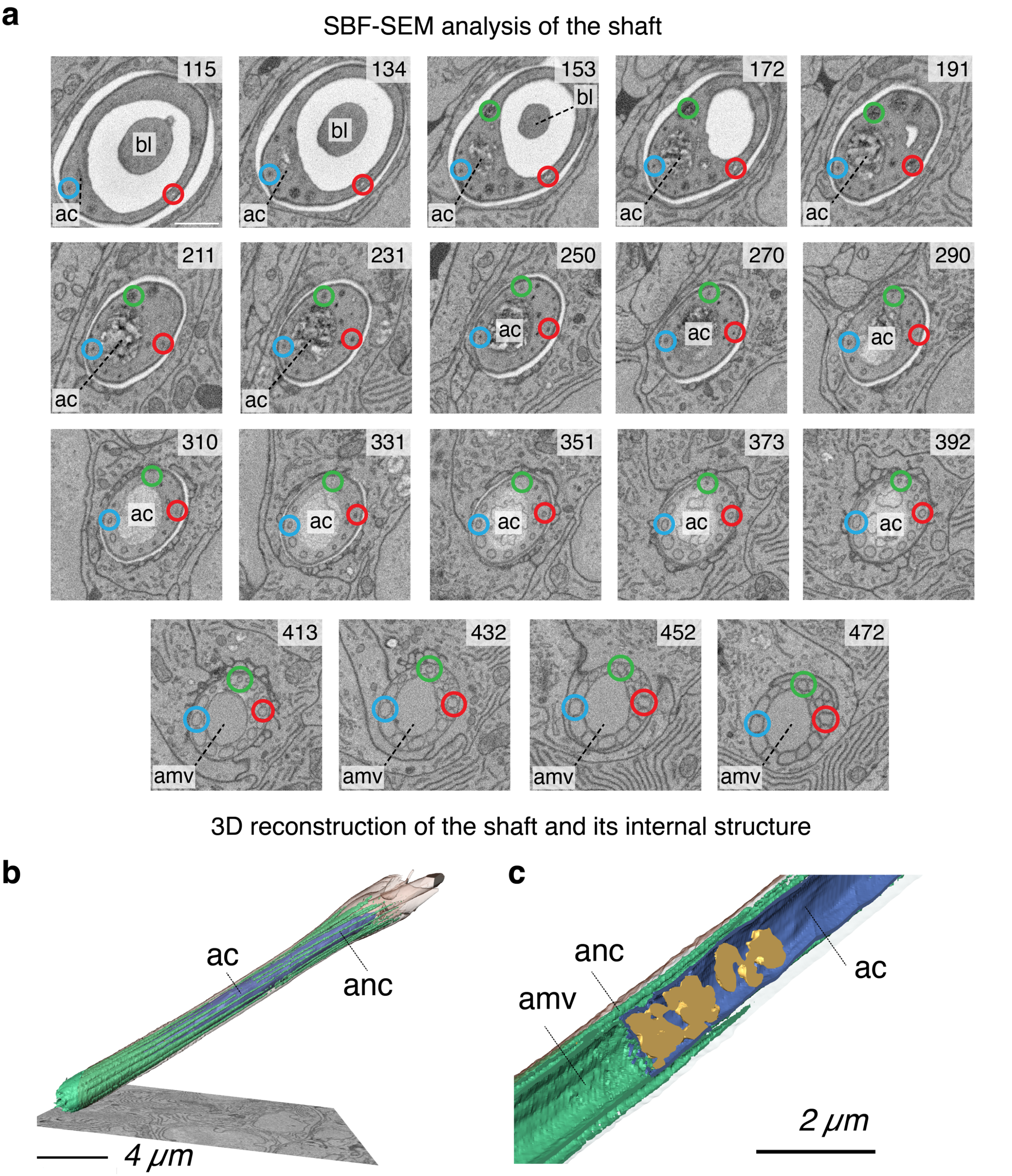

Supplementary Fig. 4. | Shaft geometry reveals dynamics in axial microvillus diameter and positional changes of annular microvilli (a) Selected SBF electronmicrographs of the shaft. Panels correspond to the indicated ~40nm cross-sections / frames of the shaft in Supplementary Video 5 and are sorted in the order of their occurrence from the distal (115) to proximal (472) direction. Consistent with Figure S1, continuity of the axial channel (ac) across sections indicates massive growth of the axial microvillus (amv) in diameter over the time of joint/ early shaft biogenesis. Tracked annular channels (red, blue, and green circles) reveal repositioning of annular microvilli from the boss of the joint to form a circumference at the base. Size bar is 1 μm. **(b, c)** Overview and details of the resulting 3D reconstruction, bl: blade (retracting); ac: axial channel; amv: axial microvillus.

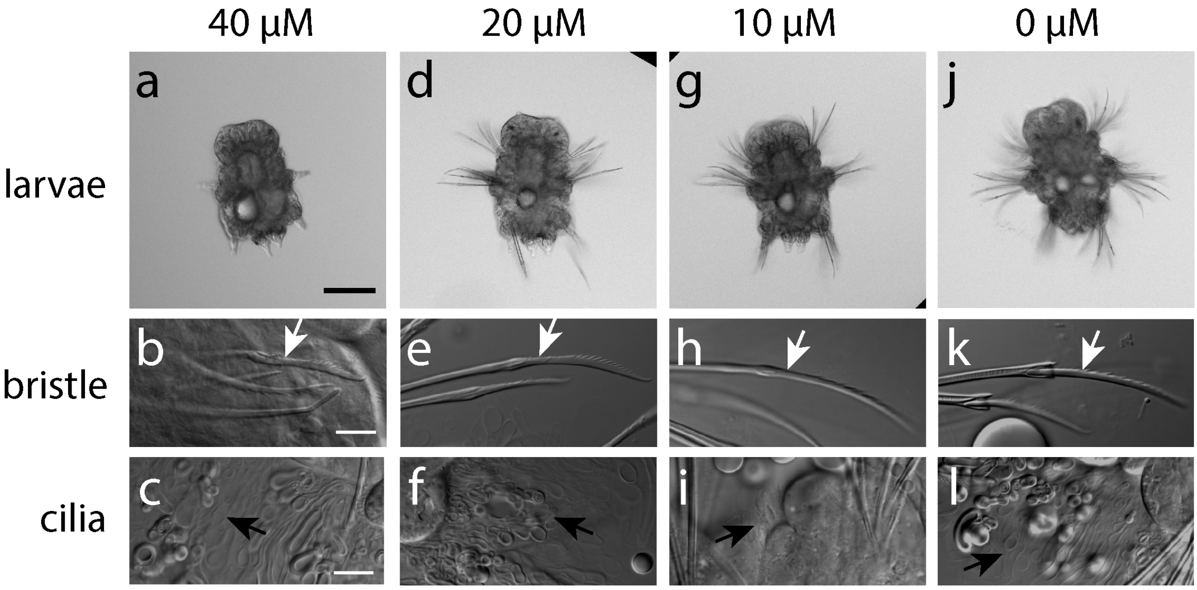

Supplementary Fig. 5 | Dose-dependence of bristle malformations upon treatment with cytochalasin D. Examples of larvae that were treated with solvent or different concentrations (40, 20, or 10 µM) of cytochalasin D from 40 hpf until 48 hpf. Larvae were then washed, and later imaged by DIC at 72 hpf. **(a-c)** A larva treated with 40 µM cytochalasin D, exhibiting normal overall morphology, but no protruding bristles (a); close-up of bristles (b) exhibiting a lack of teeth and proper joint features in the area formed during treatment (arrow); (c) the same larva exhibits normal cilia in the ciliary girdle. (**d-f**) A larva treated with 20 µM cytochalasin D, appearing normal in the overall morphology with visible protruding bristles (d), but also showing the lack teeth in proximity of the joint region, and smoother joints (arrow), while retaining normal cilia (f). (**g-i**) Similar phenotypes in a larva treated with 10 µM cytochalasin D (**j-l**) Respective vehicle control (0.01% ethanol). Scale bar in (a): 100µm.
