# Supplementary material for: Dynamic microvilli sculpt bristles at nanometric scale
Source: Nat Commun. 2024 May 13;15:3733. doi: 10.1038/s41467-024-48044-3 (PMC11091046; doi:10.1038/s41467-024-48044-3)
Supplement: Supplementary file 3 — Description of Additional Supplementary Files [file 41467_2024_48044_MOESM3_ESM.pdf]

Description of Additional Supplementary Files

File Name: Supplementary Movie 1
Description: Refractive index tomography of an isolated bristle. 3D rendering of the bristle shown in Fig 1e. Intensity corresponds to the refractive index, scale bar indicates 10 µm.

File Name: Supplementary Movie 2
Description: Cross-sections of the nascent blade. Stack of individual cross-sections trough the blade used for segmentation and reconstruction of the 3D model depicted in Supplementary Movie 3. cf. Fig 3c-f.

File Name: Supplementary Movie 3
Description: 3D reconstruction of the nascent blade and its internal structure. For representation of the different segments obtained in the 3D reconstruction, bristle material was rendered brown, and internal channels were rendered turquois. cf. Fig. 3c-f.

File Name: Supplementary Movie 4
Description: Cross-sections of the nascent joint. Stack of individual cross-sections trough the joint used for segmentation and reconstruction of the 3D model depicted in Supplementary Movie 5. cf. Extended Data Fig. 1.

File Name: Supplementary Movie 5
Description: 3D reconstruction of the nascent joint and its internal structure. As for Supplementary Movie 3, bristle material was rendered brown, and internal channels were rendered turquois. Blue colour indicates the top part of the central channel of the joint that is continuous with the axial channel of the shaft (cf. Extended Data Fig 1).

File Name: Supplementary Movie 6
Description: Cross-sections of the nascent shaft. Stack of individual cross-sections trough the shaft used for segmentation and reconstruction of the 3D model depicted in Supplementary Movie 7.

File Name: Supplementary Movie 7
Description: 3D reconstruction of a shaft and its internal structure. As for Supplementary Movie 3/5, bristle material was rendered brown, and green/blue colours indicate axial microvillus and annular microvilli/channels (green), and axial channel (blue). Segmented material in the axial channel is rendered in light brown.
